# To Duplicate Models and Impressions

**Published:** 1898-10

**Authors:** J. G. Templeton


ARTICLE VIII.

### TO DUPLICATE MODELS AND IMPRESSIONS.

J. G. TEMPLETON.

In Illinois Society.

Take printer's roller composition, melt in a water bath till dissolved. Grease the model slightly with lard, and place it the same as if to model a metal die; cover with a metal ring (a tin can opened at both ends will do), and pour the melted composition over the model. Let this stand over night. By morning the material is hardened, and the model can be withdrawn. The composition being elastic it retains its shape, and a hundred models may be poured if necessary. Printers' ink rolls are made from glue and molasses.

Having to make a full upper set of teeth, we will suppose the impression and model to have been made in the usual way. Take modeling composition, soften and flatten it out till it is about a quarter of an inch thick; press it on the model while warm, and then cut and trim to make a trial plate for the purpose of taking a bite. It should accurately fit the model. Melt a little wax around the ridge, then press a roll of softened wax on that, and trim to what you think would be a sufficient length; then try in the mouth and carefully trim the lower edge to the proper length for the teeth; if you find that it is not as desired, either add to or cut away till it is found by trying in the mouth, that the wax represents the proper length. This wax should be so cut on its articulating surface that all the lower natural teeth will strike at the same time when tried in the mouth. Now remove and soften the articulating wax surface just a little over the flame, then replace in the mouth, and do not let the patient bite into it until you have the head drawn well back, so as to put the interior muscles of the neck on a stretch; then have the patient bite a little on the wax, just to get an impression of the cusps and cutting edges of all the lower teeth. Next take an accurate impression of the lower teeth, from which make a plaster model, which will fit into the slight impressions of the teeth made in the bite taken, and then place the whole on any good articulator which can be set to maintain the relative positions. Fasten the set-screw, remove the bite, and you are ready to set the teeth to a correct articulation, and if all has been carefully done the teeth will come together without any subsequent grinding.

For an upper and lower set make trial plate of modeling composition to take the bite on, putting a piece of rather stiff wire in the lower one to stiffen it. Wax the ridges as previously described. Place a roll of softened wax on the upper trial plate, place the lower trial plate in the mouth, being careful to see that it is in its proper place, and hold it there while putting in the upper plate with the wax on it. Do not allow the patient to bite till the head is thrown back as far as you can get it; then tell the patient to bite, and keep the jaws closed till, with one finger, the wax has been well pressed on to the trial plates. Mark the center or median line on the wax. Have the patient close the lips, and then take a small straight instrument and mark on the wax the height of the lower lip. This mark should extend from one angle of the mouth to the other; you then have the line of fissure or line ot lip closure; in other words, the height of the lower lip and length of the upper to serve as a guide in making the wax-models. After thus taking the bite, place each of the models in the bite so obtained, and fasten in any good articulator; then prepare the contour wax models, which should be tried in the mouth to verify their corrections. They should come together in the mouth the same as on the articulator, and if they do not, they should be made to do so before proceeding further. Take pains to be satisfied that the wax models are correctly adjusted and give a natural expression to all the features, observing that the lower third of the face is in the proper proportion or length with the upper two-thirds, and be sure to produce the proper fullness over the region of the upper cuspids, to give as near as possible the natural contour. Then take the upper and lower plaster models off the metal articulator, and make a plaster extension to the back part of the upper model, on which place the wax models, which have heen marked while in the mouth. The lower plaster model is placed in position, and a plaster extension added to fit to that of upper plaster model. After separating these, the lower wax model is placed on the lower plaster model, and the inside space filled with wet paper, and plaster is poured over all to make the lower articulating plate to which the lower teeth are to be set. Next place the upper model in position, and set the upper teeth to the lower ones which have just been set to the lower articulating plate, and when ready for flasking; if for vulcanit plates, saw off articulating ends. In setting the teeth always set the lower teeth first. And in setting the upper teeth to the lower ones, set the bicuspids first.

Having made double sets in this way for twenty-seven years, without having to do any grinding after placing them in the mouth, the writer thinks that he has some claim to the conclusion that this method is pretty good.

3

When making an upper plate to articulate to lo natural teeth, always take an impression of the lower teeth, and in taking the bite, have the wax trimmed to show the length you wish the teeth to be, and bite into it just sufficiently to show the tips of cutting edges and cusps, where the model made from lower impression can be placed in position, etc.

To keep rubber from running between the teeth and joints in vulcanizing, after the teeth are set in the first half of the flask, the plaster trimmed and varnished, pour water on all the teeth and joints, then mix a small quantity of pure plaster, have it rather thin, and with mixing spatula cover labial and buccal surfaces, also joints, take up the piece quickly, and bring it near the mouth and blow rather sharply against the thin plaster all around, which will force it into all spaces between the teeth and blocks. After this finish flasking in the usual way and, if possible, it is well to allow the case to remain over night in the flask before packing.

To prevent plaster from adhering to the palatin surface of rubber plates, coat the model with a thick lather of soap just before packing the case.

To make rubber attachments adhere to metal plates (particularly gold), without punching holes or soldering pins and staples, clean thoroughly that portion of the plate to which the rubber is to be attached, using alcohol and chloroform to remove all wax or grease. After that scratch said portion over with a piece of new and clean sandpaper, next etch up the surface with a graver, being always careful never to touch it with the fingers or to blow the breath on it. Now with a hot spatula spread small clean piece of vulcanit over the etched surface, using considerable force till all of said portion of the plate is covered with the rubber, then finish packing the case in the usual way. When the piece is placed in the vulcanizer take half an hour to raise to 212 degrees, and from that point take three hours to raise to 320 degrees, after which allow it to cool slowly.

The greatest and most needed improvement in our profession to-day is prosthesis. In this age of progress of the arts and sciences of which so much has been said and written, and which we like to apply and hear applied to our profession, we can justfy boast of our progress in everything else.

But alas, when we see wherever we go so many evidences of the low standard of this branch of dentistry, such disfigurement, such utter failure of any artistic skill without the appearance of any effort to restore the features. These false teeth are grinning at us at every turn. False did we say? Yes, false teeth everywhere; on the street; in the car, at church and in the social gathering. Their false appearance asserts itself like a horrible ghost, while the unsuspecting victim seems to enjoy their hideousness. Many of these miserable looking so-called substitutes have the magnificent hue or shade of well-watered skim milk, and looking about as much like human teeth as a row of small white beans set in the shape of a horseshoe.

What is needed in a systematic effort to instruct the people that they may know what prosthetic dentistry can do for them. When they realize what an artistic denture is, there will be no difficulty in receiving ample compensation for it. No wonder artificial plates bring such a low price. They all look alike. Those placed in the mouths of old people being taken from the same job lot as those inserted for the lass of eighteen summers, and for this reason the more intelligent people have a perfect horror of them, and do not want to pay much for them. Any dentist who has practiced for any length of time knows what a marvelous change can be made in the features of a patient by removing an illy adapted plate, and substituting for it one well adapted in all its requirements.—*Dental Brief.*

